# Are rehabilitation services following stroke accessed equitably in Australia?: findings from the psychosocial outcomes in stroke (POISE) cohort study

**DOI:** 10.1186/1471-2458-13-884

**Published:** 2013-09-24

**Authors:** Stephen Jan, Beverley M Essue, Nick Glozier, Richard Lindley, Qiang Li, Maree L Hackett

**Affiliations:** 1 The George Institute for Global Health, PO Box M201, Missenden Road, Sydney, NSW 2050, Australia; 2 Brain and Mind Research Institute, Sydney Medical School, University of Sydney, 94 Mallett St, Camperdown, NSW 2050, Australia

**Keywords:** Rehabilitation, Equity, Access, Stroke, Young survivors, Australia

## Abstract

**Background:**

Stroke recovery is generally optimised through the provision of multidisciplinary rehabilitation. However not much is known about how equitably such services are utilised. This study examines the determinants of physiotherapy and speech therapy utilisation in rehabilitation within a cohort of young stroke survivors in Australia.

**Methods:**

Psychosocial Outcomes in StrokE (POISE) was a three-year prospective observational study involving stroke survivors between the ages of 18 and 65 years recruited within 28 days of stroke. It was conducted in 20 stroke units in Australia. Participants were interviewed at 28 days (baseline), 6 and 12 months after stroke about their demographic and socioeconomic background, economic and health outcomes and the use of services. The primary outcome in this paper is utilisation of rehabilitation in the 12 months after stroke.

**Results:**

Of 414 participants, 254 (61%) used some rehabilitation in the 12 months post stroke. The strongest predictor of use of these rehabilitation services was dependency at 28 days, as assessed by need for assistance in activities of daily living (OR=33.1; p<0.0001). Other significant variables were two dimensions of social capital - an individuals’ ability to make important decisions, which had a negative relationship (OR = 0.43; p=0.04) and number of close friends (OR= 1.042; p=0.02).

**Conclusion:**

These findings demonstrate that socio-demographic factors exert little influence on the use of rehabilitation services in working age stroke patients and that the use of such services is primarily determined by 'need’. Such findings suggest that services are being provided equitably.

**Trial registration:**

ANZCTRN12608000459325

## Background

Stroke has potentially catastrophic economic and social consequences on individuals and their families [1,2]. A key element in achieving optimal recovery, returning to work and maintaining good quality of life, is the provision of multidisciplinary rehabilitation, with such services recommended in clinical guidelines in Australia [3,4] and internationally [5-8]. For those with mild to moderate disability, in particular, rehabilitation is recommended alongside early discharge [3].

Inpatient rehabilitation, initiated rapidly after stroke through multi-disciplinary teams within dedicated stroke units, represents the option with the strongest evidence base and thus is considered gold standard care for recovery in the post acute phase [3,9]. Unfortunately in Australia, as in most other countries, the availability of this type of specialised service following stroke is limited. In many settings, the alternative within an inpatient setting is treatment within a general ward. In relation to outpatient rehabilitation, a systematic review in 2004 indicated that such rehabilitation within 1 year post stroke improves activities of daily living and reduces deterioration [10]. The organisation and funding of such services however varies by setting - in Australia outpatient services may be accessed privately and either subsidised through private health insurance or paid directly out of pocket. A limited publicly funded option also exists where rehabilitation is accessed and co-ordinated through general practitioner referrals and subsidised through Medicare.

Despite the multiplicity of avenues with which such services can be accessed, not much is known about whether, in a community setting, the use of rehabilitation services in Australia is equitably distributed. Whilst equality of access to hospital and primary care is seen as the cornerstone of the health care system in Australia, it is less clear whether this principle extends to allied health and rehabilitation. Indeed it is well recognised that the availability of rehabilitation/allied health services tends to be concentrated geographically in the higher socioeconomic and metropolitan areas [11]. Given such disparities, and the potential cost barriers faced by patients particularly in an outpatient setting, systematic inequities potentially exist in access to rehabilitation. In this study we examine whether these services are accessed equitably by determining the extent to which 'need’ relative to socio-demographic factors such as rurality, socioeconomic status and private health insurance status influences the utilisation of rehabilitation services.

## Methods

### Design and overview

The Psychosocial Outcomes in Stroke (POISE) was a three-year prospective observational study that consecutively recruited English-speaking individuals (or their proxy) between the ages of 18 and 65 years within 28 days of stroke. It was conducted in 20 stroke units in Australia [1,12,13]. Consented participants were interviewed via telephone at 28 days, 6 and 12 months after stroke. Data were collected on demographic and socioeconomic background, economic outcomes, quality of life, mood, social contacts, stroke type, level of disability and use of rehabilitation services. The Human Research Ethics Committee of the Sydney South West Area Health Service approved the study and written informed consent was obtained from all participants or their proxy.

### Outcomes

The main outcome in this study was whether a participant used physiotherapy or speech therapy or both in the 12 months after stroke.

### Variables of interest

The main variable of interest was dependency at 28 days, which was measured as a proxy of need. It was assessed based on a single item in which respondents indicated whether they were dependent on another member of their household for help with everyday activities (e.g. self care: dressing, showering, feeding; to move around; for communication activities).

Socio-demographic variables included age, sex, job type, income, urban/rural place of residence, occupational class, private health insurance status, income protection insurance status and living arrangements.

In addition a number of illness-related variables were investigated including co-morbidity (Charlston Co-morbidity Index) [14], stroke type, cognitive status (Telephone Interview for Cognitive Status (TICS) [15]) at 28 days and depression (Hospital Anxiety and Depression Scale (HADS) [16]) at 28 days.

Social contact and empowerment variables were individual questions extracted from the World Bank Integrated Questionnaire for the Measurement of Social Capital (SC-IQ) [17] and involved questions about number of close friends, neighbourhood trust and empowerment.

### Statistical analysis

Univariate analyses were used to determine the individual associations between the main outcome and various pre-specified baseline characteristics. Multivariate logistic regression was then conducted to identify and estimate the predictors of this outcome after stroke. The model was specified through backward elimination using a threshold of p=0.2. Data analyses were conducted using SAS version 9.2.

## Results

**Figure 1:**
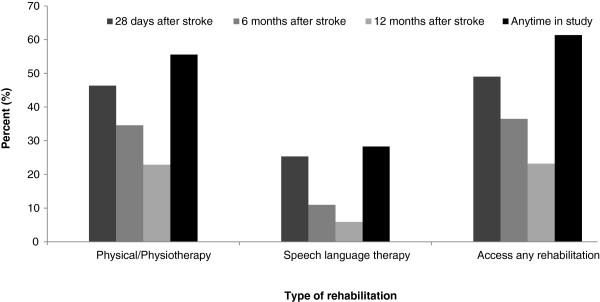
Percentage of patients using rehabilitation services at 28 days, 6 and 12 months post-stroke (separate file).

440 participants consented to involvement in the study, but 26 of these were unable to provide a baseline assessment. 414 participants were included in this study. Figure 1 provides details of the utilisation of defined rehabilitation services at 28 days, 6 months, 12 months and at any time during follow-up. Of the 414 participants, 254 participants (61%) used some rehabilitation in the 12 months post stroke, with 230 (56%) accessing physical/physiotherapy and 117 (28%) using speech therapy.

Table 1 shows the baseline and post-stroke characteristics of the study population according to whether or not they utilised any rehabilitation. It indicates that 71 out of 74 (96%) individuals who reported dependency at 28 days had accessed rehabilitation at some time in the 12 months following stroke.

**Table 1:** Characteristics of participants by whether they utilised rehabilitation services post-stroke

**Rehabilitation after stroke**			
	Yes n=254 (%)	No n=160 (%)	Univariate p-value
*Demographic information*			
Age, mean (±SD)	52.8 (9.8)	51.6 (10.4)	0.22
Male	163/254 (64)	117/160 (73)	0.06
Education:			
School certificate or less	97/251 (39)	54/160 (34)	0.25
HSC/trade certificate	68/251 (27)	43/160 (27)	0.57
Diploma/degree or higher (ref)	86/251 (34)	63/160 (39)	
Married/defacto	159/254 (63)	106/160 (66)	0.12
Cognitive status score ( TICS <21)	44/220 (20)	21/154 (14)	0.11
Living alone	45/254 (18)	24/160 (15)	0.47
*Medical history*			
Smoker	114/253 (45)	63/160 (39)	0.26
Hazardous drinking level (score >8 on AUDIT-C)	38/253 (15)	27/160 (17)	0.61
Co-morbidity (Charlson Comorbidity index)	141/253 (56)	67/159 (42)	0.01
Dependent in ADLs before stroke	6/253 (2)	2/159 (1)	0.43
Previous treatment for depression	98/254 (39)	53/160 (33)	0.26
*Economic circumstances*			
Employment (pre-stroke)			
Full-time/part-time (ref)	156/254 (61)	115/160 (72)	
Retired/unemployed	98/254 (39)	45/160 (28)	0.03
Manual occupation (pre-stroke)	123/230 (53)	74/153 (48)	0.33
Income			
Low (<AUD$600 per week)	49/226 (22)	25/147(17)	0.16
Middle (AUD$600 to AUD$1000 per week)	38/226 (17)	28/147 (19)	0.91
High (>AUD$1000 per week) (ref)	109/226 (48)	83/147 (56)	
No private health insurance	138/251 (55)	82/159 (52)	0.50
No income protection insurance	227/251 (90)	137/159 (86)	0.18
Carer payment/allowance	86/252 (34)	44/159 (28)	0.17
Economic hardship at baseline	91/254 (36)	59/160 (37)	0.83
Main earner in household	144/249 (58)	100/159 (63)	0.31
*Social capital*			
Believes they have the power to make important decisions	187/247 (76)	138/159 (87)	<0.01
Likely to have access to someone beyond close relatives willing and able to lend one week’s wages	199/247 (90)	132/156 (85)	0.30
Agree that most people in their neighbourhood are willing to help	195/251 (78)	111/159 (70)	0.07
Believes they need to be alert to potential harm in their neighbourhood	74/249 (30)	53/158 (34)	0.42
Number of telephone calls (week) (±SD)	30.4 (29.1)	32.2 (28.7)	0.55
Number of close friends (±SD)	9.5 (11.8)	8.1 (10.4)	0.22
Number of times got together with family/friends since stroke	3.5(6.0)	4.6 (5.4)	0.05
AFTER STROKE (28 days)			
Stroke sub-type			
Ischaemic (ref)	209/254 (82)	136/160 (85)	
Intracerebral haemorrhage	32/254 (13)	15/160 (9)	0.67
Subarachnoid haemorrhage	2/254 (1)	2/160 (1)	0.63
Unknown/missing	11/254 (5)	7/160 (5)	0.39
Dependent in activities of daily living at 28 days	71/232 (31)	3/154 (2)	<0.01
Returned to any paid work	23/155 (15)	52/113 (46)	<0.01
Depression at 28 days (HADS depression subscale >= 8)	36/218 (17)	17/154 (11)	0.14
Anxiety at 28 days (HADS anxiety subscale >= 8)	55/218 (25)	37/154 (24)	0.79

**Figure 2:**
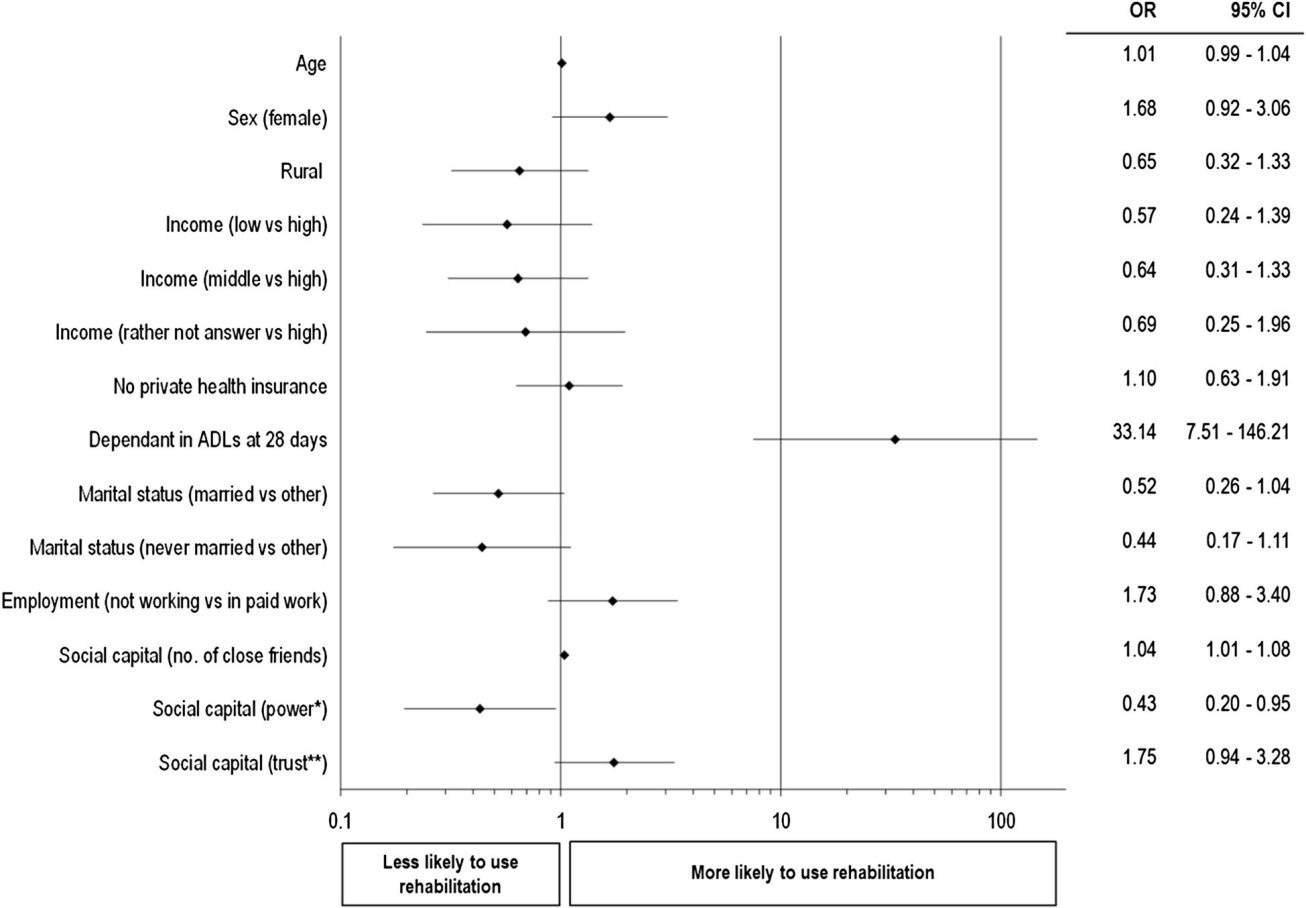
Multivariate analysis: predictors of the use of rehabilitation services post-stroke (separate file).

The multivariate analyses indicate that the strongest predictor of use of rehabilitation services was dependency at 28 days (OR=33.1; p<0.01). Other significant variables were two separate dimensions of social capital - respondents’ power to make important decisions, with those responding 'yes’ having significantly lower odds of using physiotherapy and/or speech pathology in the 12 months post stroke (OR=0.43; p=0.04) and the number of close friends reported by respondents, with each additional friend increasing the odds of using rehabilitation by 4.2% (OR= 1.042; p=0.02). The variables included in the model that were not found to be statistically significant were age, sex, marital status, rurality, health insurance status or income, job type and a third dimension of social capital relating to neighbourhood trust (see Figure 2).

## Discussion

The participants encompass individuals well-represented across socioeconomic groups, age categories, gender, stroke type and level of illness/disability. The findings indicate that in the 12 months post stroke, physiotherapy services were used by 55% of younger survivors, speech therapy by 28% and either of such services by 61%. 96% of those reporting initial disability at 28 days after stoke accessed these rehabilitation services. Disability was the strongest predictor of such use. Other factors that were significant were self-perceptions about the level of power over one’s life decisions, which was negatively associated with use of rehabilitation; and social contacts, as defined by the number of close friends reported, which had a positive relationship.

That self-reported disability at 28 days strongly predicted use of rehabilitation services is encouraging. It suggests that these services are being used according to the extent to which they are needed. This is at odds with some evidence from Canada, where access to rehabilitation has been found to differ by functional status, favouring those with milder impairment [18]. Indeed the Ontario Stroke Evaluation Report 2011 citing evidence that the proportion of those with severe disability admitted to rehabilitation had been declining between 2003 and 2010 (from 36.7% to 31.9%), recommended that the barriers to care to those with more severe impairment be identified and addressed [18,19].

Further encouragement can be gained from the findings of this study by the lack of significant effect of socioeconomic and socio-demographic variables, as defined by a variety of measures including income and job type, health insurance status, age and rurality. These findings in concert suggest 'need’ as being the primary determinant in the use of these rehabilitation services in stroke patients and that access to care is not differentiated by socio-demographics. They are consistent with the premise that rehabilitation services are being provided according to the established equity objective of 'equal use for equal need’.

The evidence in relation to this issue internationally is mixed. In the UK, audit data suggests that access to post-stroke rehabilitation is inequitably distributed [20] with substantial variation in use observed across regions [21]. A review of the US literature found no evidence of racial-ethnic disparities in access to post stroke rehabilitation, both in terms of use of services and time to admission [22]. Indeed within the Veterans’ Affairs system there is some evidence that African-Americans are more likely to access such services [23,24].

One limitation of the study was that it examined only a subset of the suite of rehabilitation services potentially available to patients. Rehabilitation is most effective as a multidisciplinary activity including social work, occupational therapy, specialist nurse support, family care worker, mental health worker and case management. However, the involvement of physiotherapy and to some extent speech therapy in such multidisciplinary care is often integral, and as such, may be viewed as a marker for access more generally to appropriate rehabilitation. The limitation here is that access to rehabilitation has been assessed as a dichotomous outcome; future research should examine in more detail the nature, timing and level of rehabilitation received as these factors highly influence the outcomes of such services [20].

As the study was undertaken in a working age population, it is not possible to generalise to older stoke survivors who, due to the availability of age-related services and government health care concessions, face challenges in accessing care that are very much different.

The current study applied the widely-used SC-IQ to measure the influence of social capital. Taken at face value the findings indicate that those with greater empowerment tend to eschew rehabilitation, whilst the number of social contacts increases the propensity for patients to access such services. These findings provide at least tentative support for peer and family support programs as avenues for encouraging the use of rehabilitation services. Further research would examine in more detail the influence of social networks on utilisation of services and identify ways in which health sector programs can capitalise on these existing structures to promote access to care.

## Conclusion

The study finds no evidence of systematic inequities in utilisation of rehabilitation services in the 12 months after stroke in a younger survivor population and that the use of such services is overwhelmingly influenced by patient need. It suggests that at least in terms of post-stroke rehabilitation services in Australia, access to care is equitably distributed.

## Competing interests

The authors declare that they have no competing interests.

## Authors’ contributions

SJ conceived the original idea for the paper and wrote the first draft of the manuscript. MH designed and was the lead investigator on the POISE study. QL undertook the statistical analysis. All authors (SJ, BE, NG, RL, QL, MH) contributed to the running of the POISE study, commented on drafts of the manuscript and were involved in interpreting the findings. All authors read and approved the final manuscript.

## Pre-publication history

The pre-publication history for this paper can be accessed here:

http://www.biomedcentral.com/1471-2458/13/884/prepub
